# The weight of flash chromatography: A tool to predict its mass intensity from thin-layer chromatography

**DOI:** 10.3762/bjoc.12.228

**Published:** 2016-11-08

**Authors:** Freddy Pessel, Jacques Augé, Isabelle Billault, Marie-Christine Scherrmann

**Affiliations:** 1 Université Paris Sud, ICMMO, UMR CNRS 8182, Bâtiment 420, 91405 Orsay Cedex, France; 2 Université de Cergy-Pontoise, LCB, EA 4505, 5 mail Gay-Lussac, Neuville sur Oise, 95031 Cergy-Pontoise, France

**Keywords:** environmental factor, flash chromatography, green metrics, mass intensity, purification

## Abstract

Purification by flash chromatography strongly impacts the greenness of a process. Unfortunately, due to the lack of the relevant literature data, very often this impact cannot be assessed thus preventing the comparison of the environmental factors affecting the syntheses. We developed a simple mathematical approach to evaluate the minimum mass intensity of flash chromatography from the retention factor values determined by thin-layer chromatography.

## Introduction

As part of a more respectful environmental chemistry, many efforts have been made to reduce the impact of chemical transformations by developing high atom-economic reactions, alternative reaction media or high-performance catalysts. The formation of a pure chemical product not only requires reactants, solvents, promoters and catalysts used in the reaction, but also other materials used for the work-up and for the purification steps. The Sheldon *E* factor [1–2] and the mass intensity *MI* [3–5], which are defined according to Equation 1 and Equation 2, respectively, are classical metrics based on the economy of material for evaluating the greenness of a process.

It is worth noting that these mass-based metrics allowed to quantify the mass of waste but did not take into account their potential for negative effects on the environment. These two metrics are related by Equation 3 [6].

[1]
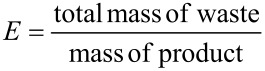


[2]
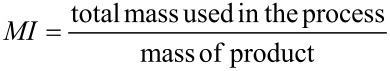


[3]
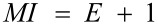


The amount of waste includes the amount of the byproducts, but also the amount of non-reacting starting materials, auxiliaries, catalysts or any additives such as acids, bases, salts, solvents of the reaction or solvents required for the work-up and the purification. We demonstrated that the mass intensity could be easily calculated for linear and convergent sequences from the global material economy GME (Equation 4), which is related to the atom economy, the yields of each step, the excess of reactants and the mass of auxiliaries [6–7].

[4]
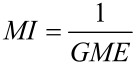


It can be fractioned into three parts: reaction itself (*MI*_R_), work-up (*MI*_W_) and purification (*MI*_P_) as shown by Equation 5 [8].

[5]
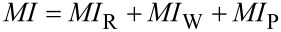


Any value of the *E* factor which does not take into account the work-up and purification steps is nonsensical, since the values of *MI*_W_ and *MI*_P_ are often much higher than the value of *MI*_R_.

In order to compare the greenness of different processes, each term of Equation 5 has to be known. From the literature data it is possible to retrieve information concerning the amount of reactants, solvents and catalysts allowing the calculation of *MI*_R_. Moreover, since the work-up is usually well described, it is easy to gain access to *MI*_W_. In contrast, the amount of auxiliaries and solvents used in the purification of products is very often omitted. For example, the mass of silica gel and eluents used are never mentioned, which prevents the reader from calculating *MI*_p_, and thus having the actual value of the *E* factor. The impact of chromatography on sustainability was recently discussed [9] and we propose here a method to evaluate such an item. This tool can also allow the chemist to evaluate, from a thin-layer chromatography (TLC), the minimum mass required to perform a flash chromatography. Our calculations are based on the preparative chromatographic technique largely used by chemists [10–12] and on our own experiments.

## Results and Discussion

The publication of Still et al. [10] describing flash chromatography in 1978 greatly facilitated the post synthesis purifications which were, until then, often carried out by gravity column chromatography that was time consuming and did not always lead to effective separations. Since then, various automated systems equipped with pumps and eventually detectors and using disposable pre-packed silica cartridges were marketed offering great ease of use.

The mass intensity of purification by chromatography (*MI*_Chr_) is the ratio between the total mass used to perform the chromatography (i.e., the sum of the mass of silica (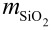) and the mass of eluent (*m*_eluent_)) and *m*_p_, the mass of the product (Equation 6).

[6]
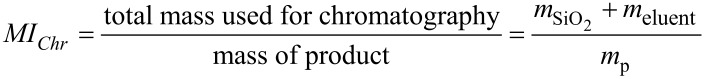


### Mass of silica

The size of the column for chromatography and therefore the amount of silica and solvent depends on the mass of the sample and on the difficulty of separation of the products. This difficulty may be evaluated by Δ*R*_f_ that is the difference between the retention factor *R*_f_ of products in TLC (thin-layer chromatography). Based on their experimentations, Still et al. recommended typical column diameters (constant height) and sample loading for difficult separations (0.2 > Δ*R*_f_ ≥ 0.1) or more easier separations (Δ*R*_f_ ≥ 0.2) [10]. Using a column height of 5.9 inches (ca. 15 cm) and considering that the silica has a density of 0.5, correlations have been established between the mass of silica to be used and mass (*m*_s_) of the sample to be purified (Table 1, entry 1) [12]. For commercial pre-packed cartridge indications are also provided [13–15] and we have selected some data to obtain a general trend (Table 1).

**Table 1:** Mass of silica (in grams) to be used depending on the mass of sample to be purified for manually packed columns and some commercial pre-packed cartridges.

Entry	Cartridge	Particles shape	Average particle size (μm)	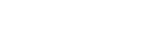		

				difficult separation	moderately difficult separation	easy separation

1	Silica gel^a^	irregular	40–63	151.2 *m*_s_ + 0.5	59.8 *m*_s_	
2	RediSep^TM^	irregular	35–70	1000 *m*_s_	25 *m*_s_	14. *m*_s_
3	EasyVario Flash^TM^	irregular	15–40	33.3 *m*_s_		
4	SNAP^TM^	irregular	40–50	10 *m*_s_	20 *m*_s_	10 *m*_s_
5	SNAP Ultra^TM^	spherical	25	50 *m*_s_	10 *m*_s_	5 *m*_s_

^a^Manually packed glass column.

The mass of silica required to purify *m*_s_ g of sample may therefore be estimated by Equation 7. Excluding the equation obtained for difficult separations with the RediSep^TM^ cartridge leading to extremely high values of mass of silica (Table 1, entry 2), and partially the equations obtained with spherical silica (SNAP Ultra^TM^, Table 1, entry 5), the values of *A* range from 10 to 152.

[7]
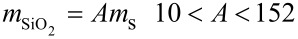


### Mass of eluent

**Figure 1:**
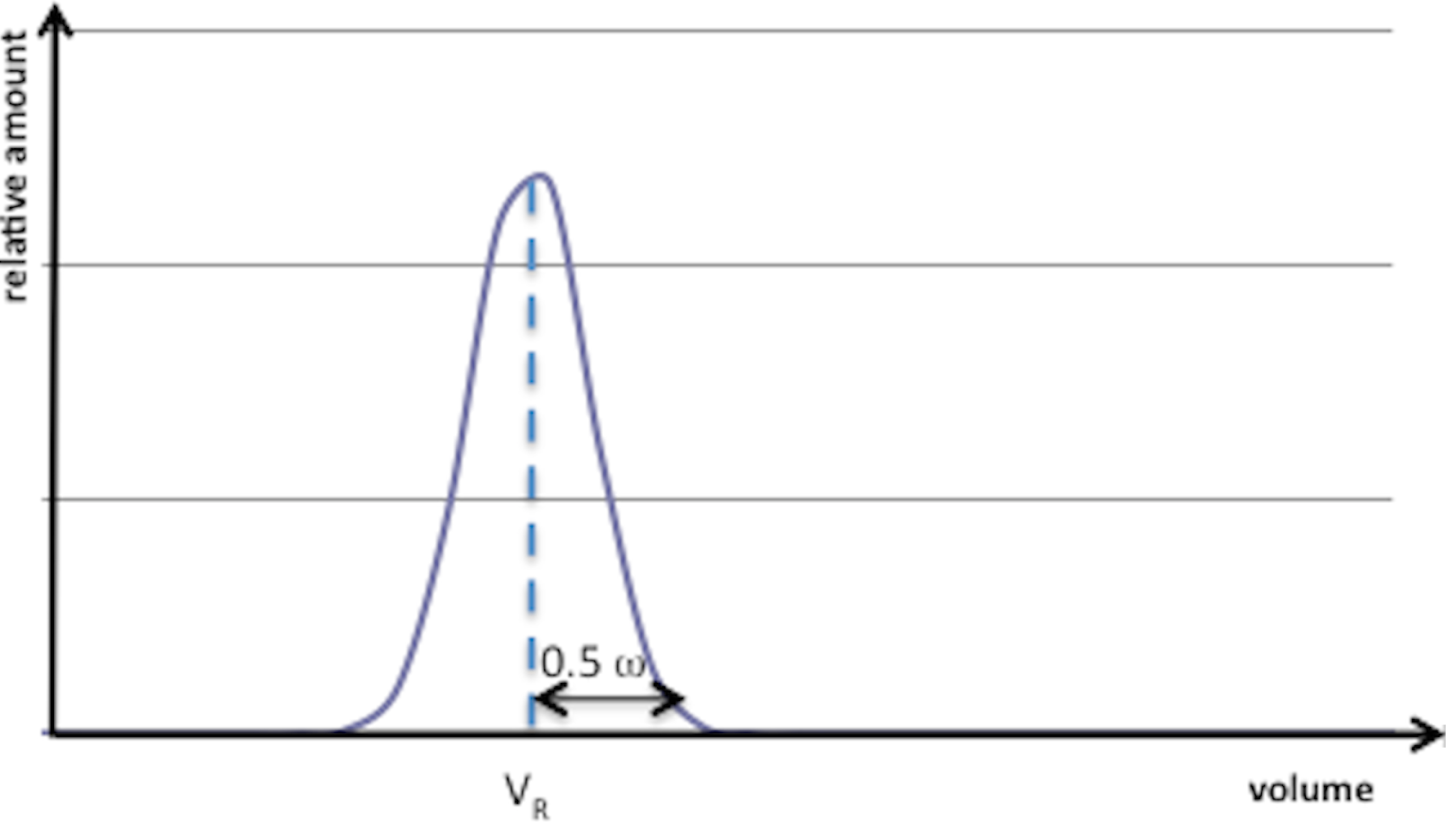
Chromatographic peak of a compound eluted at a retention volume *V_R_* with a width ω.

The total amount of solvent required for carrying out a chromatography is composed of the part used to pack the column, of that needed to elute the sample, (i.e., the retention volume *V*_R_ and the half width of the chromatographic peak ω (Figure 1)), and the void volume *V*_0_ that corresponds to the mobile phase volume in the packed column.

Considering that the solvent used to pack the column is generally recycled, the volume of eluent required can then be expressed by Equation 8.

[8]
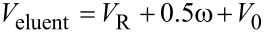


Under ideal conditions, the retention volume *V*_R_ can be related to the *R*_f_ by Equation 9.

[9]
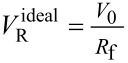


Some deviations of this equation were observed for silica gel column and a correction factor C was proposed [12], so that *V*_R_ should be calculated using Equation 10. A value of 0.64 was found for manually packed columns, while for commercial cartridges, the value of *C* was 0.66.

[10]
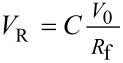


The half width of the chromatographic peak can be estimated by assuming that the peak is described by a Gaussian with a standard deviation σ (Equation 11).

[11]
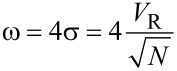


In this equation, the term *N* represents the efficiency of the chromatographic column, i.e., the system's ability to elute the same compounds at identical rates in order to obtain thin peaks. *N* is defined as the number of theoretical plates of the column.

Using Equations 8,10 and 11, the mass of eluent can be expressed by:

[12]
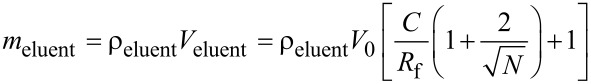


The void volume *V*_0_ is connected to the column volume *V*_C_ by the porosity of the silica 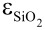 (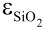 = 0.9) and the volume of the column depends on the mass and density (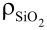 = 0.5) of the silica according to Equation 13 and Equation 14.

[13]
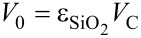


[14]
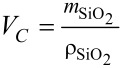


We can then deduce the following equation for the mass of eluent:

[15]
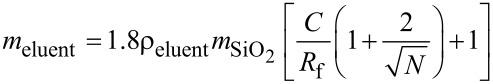


Although *N* depends on various parameters such as the size of the column, the packing particles, the quality of the packing and the flow of the mobile phase, an average value of 35 was proposed for flash chromatography column [16]. Alternatively, in order to take into account broadening of the chromatographic peaks due to the amount of compounds in the sample, it was proposed [12] to evaluate *N* as a function of the mass fraction of the product in the sample (*m*_P_ = *xm*_s_), for difficult separation (Equation 16, B = 51.70) or more easier separation (Equation 16, B = 33.64).

[16]
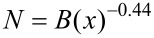


### Mass intensity of a chromatography

As already stated above, the mass intensity of purification by chromatography is the ratio between the total mass *m*_T_ used to perform the chromatography and the mass *m*_P_ of the product (Equation 6). The total mass is the sum of the silica and eluent masses that can be expressed from Equation 7 and Equation 15.

[17]
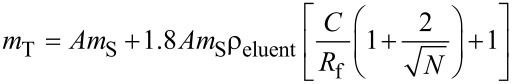


Considering x, the mass fraction of the product in the sample the theoretical expression of *MI*_Chr_ becomes:

[18]
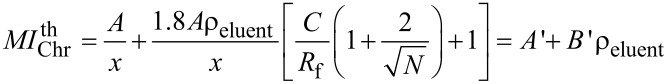


### Application

**Scheme 1:**
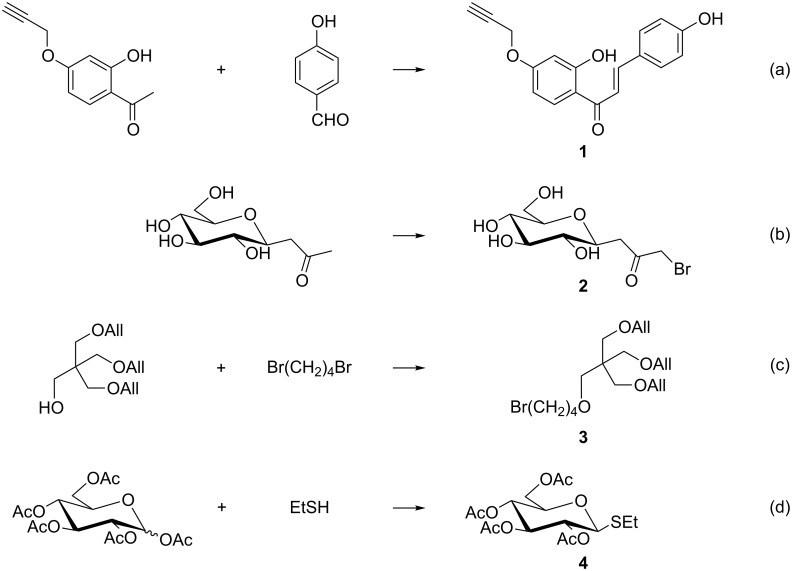
Reactions used as examples. (Substrates and products, all the reagents are not shown).

We chose 4 syntheses whose crude reaction products were purified by flash chromatography to illustrate the calculations developed above (Scheme 1).

In all cases, *C* was set at 0.64 and 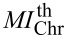
was calculated using *N*_calc_ (Equation 16) or *N* = 35. The value of 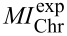 was determined according to the experimental data.

Compound **1**, obtained by aldol condensation (Scheme 1, reaction a) in 80% yield [8], was chromatographed on a manually packed column using as eluent a 7:3 cyclohexane–acetone mixture (Table 2, entry 1). The mass fraction of product in the crude reaction mixture (79%) was calculated after chromatography taking into account the isolated mass of **1**. The values calculated using Equation 18 with *N* = 35 or *N*_calc_ (Equation 16, *B* = 33.64 or *B* = 51.70) deviated only from 6, 7 and 11% of the experimental value, respectively. Another experiment (a(bis), Table 2, entry 2) led to a crude reaction mixture containing 60% by weight of **1** which was purified using a disposable cartridge (PuriFlash SIHP 30 µm, Interchim) and cyclohexane–AcOEt (7:3) as the eluent. Also in this case, the calculated values were very close to the experimental ones (differences of 1, 3 or 6%).

**Table 2:** Comparison of the experimental values of the mass intensity of chromatography (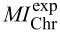) with the theoretical estimated values (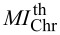) for various reactions (Scheme 1).

Entry	Reaction	*R*_f_	*A*	*x*	*A’*	ρ_eluent_	*N*	*B’*	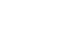	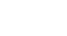

1	a	0.1	49	0.79	62	0.78	35 37^b^ 57^c^	1072 1065 1019	901 896 860	962
2	a(bis)^d^	0.15	47	0.60	78	0.81	35 42^b^ 65^c^	946 928 892	843 829 800	857
3	b	0.13	30	0.38	79	0.89	35 51^b^ 79^c^	1074 1033 995	1031 994 961	1161
4	c	0.30	20	0.42	47	0.65	35 49^b^ 76^c^	324 314 304	258 252 245	250
5	d	0.20	30	0.61	49	0.81	35 42^b^ 64^c^	468 459 442	427 421 407	458

^a^Calculated with the exact values and not with the rounded off numbers *A’* and *B’*. ^b^Calculated using Equation 16 with *B* = 33.64; ^c^Calculated using Equation 16 with *B* = 51.70. ^d^Reaction a, other experimental conditions.

**Figure 2:**
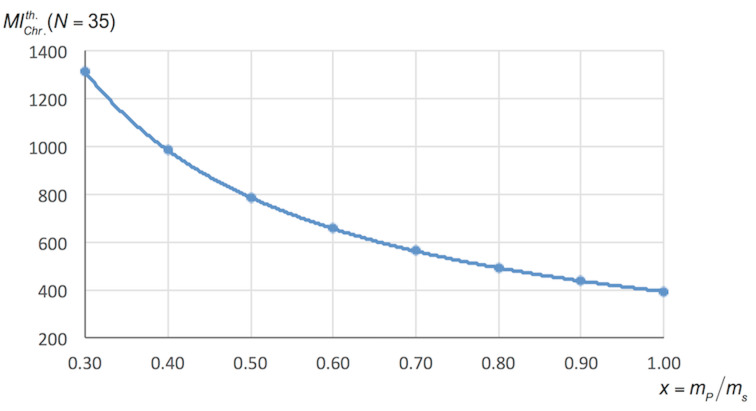
Variation of 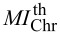 with *x* for reaction b (Scheme 1).

The crude mixture of reaction b, a bromination in alpha position of a ketone leading to **2** [17–18] in 54% yield, was chromatographed using AcOEt–MeOH (9:1) as the eluent [8]. The mass fraction of compound **2** in the sample was only 38%, leading to high value of 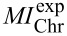 (Table 2, entry 3). The calculations lead to 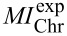
values having differences of 11, 14 and 17% compared to the experimental value. Obviously the lower is the proportion by weight of the compound in the sample, the higher is the mass intensity for the chromatography. This variation in (1/*x*) was represented for reaction b in Figure 2. Therefore when this proportion is not precisely known, which is the most frequent case before performing the purification, it is possible to estimate a minimum value of *MI*_Chr_ setting *x* = 1, or, if the mass of the sample to be purified is higher than the theoretical mass of product, *x* can be calculated assuming a 100% yield (Equation 19).

[19]
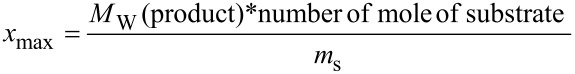


It is also clear that if a treatment (e.g. extraction) can reduce the mass of the sample to be purified, it would reduce the mass intensity related to chromatography. This should obviously not be to the detriment of the overall mass balance.

This can be illustrated by example c (Scheme 1). In fact, this alkylation reaction was carried out in the presence of a large excess (4 equiv) of dibromobutane to get compound **3** in a good yield (73%) [19]. Some of this excess was removed from the crude reaction product by distillation, reducing the mass of the sample by 53%. This allowed to recycle the reactant but also to greatly reduce the weight of silica to be used (*A* = 20) and, accordingly the mass of solvent (Table 2, entry 4). This purification with a particularly low *MI*_Chr_, compared to the other examples, corresponded to a filtration on silica gel rather than to a flash chromatography.

The last example (Scheme 1d) is a *S*-glycosylation (isolated yield = 62%) leading to compound **4** [20]. For this crude reaction mixture containing 61% of **4**, a correct separation was obtained on TLC with the mobile phase cyclohexane–EtOAc (75:25). Again, the values obtained by the calculation were close to the experimental ones, with differentials of 7, 8 and 11% depending on the value taken for *N* (Table 2, entry 5).

**Figure 3:**
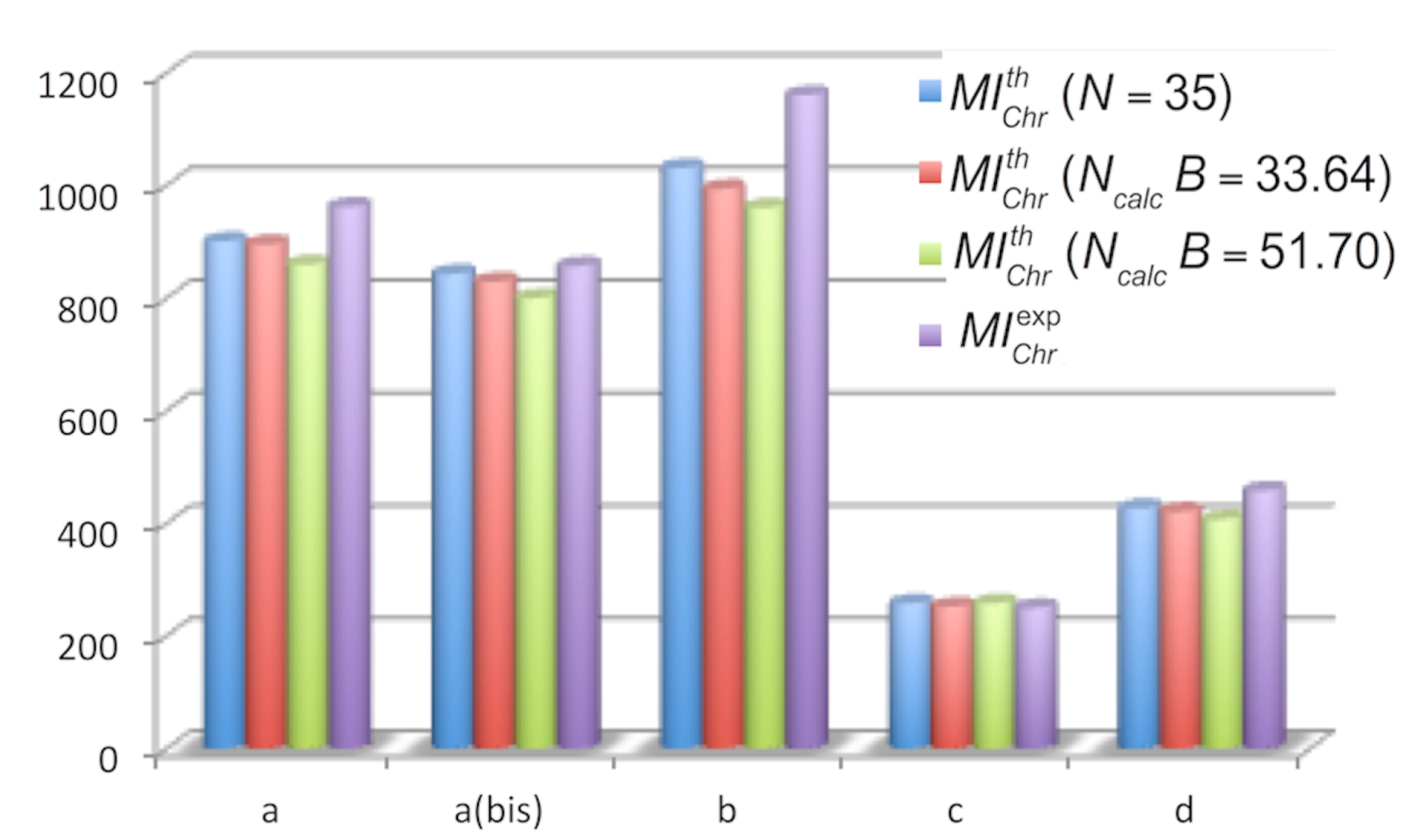
Comparison between calculated and experimental values of *MI*_Chr_ for the reactions of Scheme 1.

In each case, the calculation afforded values close (deviations <17%) to the experimental value (Figure 3). As already pointed out above, the calculation depends on the value of *x* that it is not always easy to estimate, but it is possible to estimate a minimum of the mass of intensity related to the chromatography by setting *x* closed to 1.

**Figure 4:**
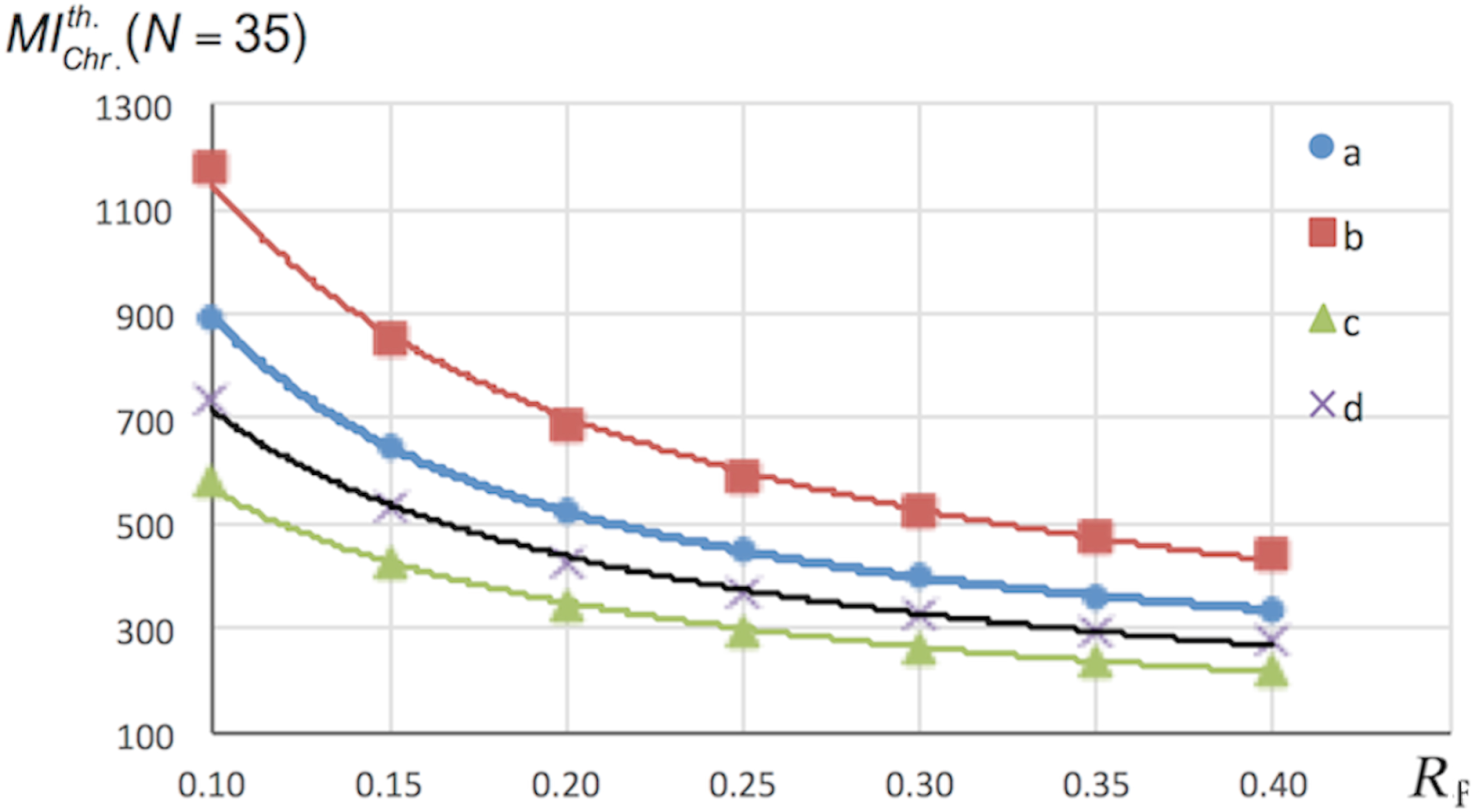
Variation of 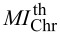 (*N* = 35) with *R*_f_ for the reactions of Scheme 1.

The value of *MI*_Chr_ also depends on the retention factor (*R*_f_), especially when the latter is less than 0.2 (Figure 4). An estimation of the minimum is also possible by setting an *R*_f_ to a value close to 0.35, as recommended in the seminal paper of Still et al. [10].

## Conclusion

If the impact of chromatography on the environmental factor *E* of a process seems pretty obvious, we have developed here a tool to quantify it. In an extremely favourable case with a 95% pure sample (*x* = 0.95), a very easy separation achievable with a small mass of silica (A = 10) and *R*_f_ = 0.35, we find, for low density eluent (0.6), an *MI*_Chr_ value close to 50. By doubling the amount of silica, which is closer to reality, the *MI*_Chr_ value is about 100. In real cases chosen here as examples, we have shown that the values were fairly between about 200 and 1200.

Since it is clear that chromatography should be avoided wherever possible, works proposing alternative purification methods have been published [9,21–22]. When the purification by flash chromatography is necessary, solvents with low environmental impacts should be used [23–25]. In this context, super critical chromatography which allows to obtain very low retention volumes and easy recycling offers an interesting alternative [26] but requires a significant investment.
